# Applying the technique of volume‐modulated arc radiotherapy to upper esophageal carcinoma

**DOI:** 10.1120/jacmp.v15i3.4732

**Published:** 2014-05-08

**Authors:** Pan Ma, Xiaozhen Wang, Yingjie Xu, Jianrong Dai, Luhua Wang

**Affiliations:** ^1^ Department of Radiation Oncology Cancer Institute & Hospital, Chinese Academy of Medical Sciences Beijing China

**Keywords:** volumetric‐modulated arc therapy, intensity‐modulated radiation therapy, simplified intensity‐modulated radiation therapy, upper esophageal carcinoma, saving time

## Abstract

This study aims to evaluate the possibility of using the technique of volume‐modulated arc therapy (VMAT) to combine the advantages of simplified intensity ‐ modulated radiation therapy (sIMRT) with that of regular intensity‐modulated radiation therapy (IMRT) in upper esophageal cancer. Ten patients with upper esophageal carcinoma were randomly chosen in this retrospective study. sIMRT, IMRT, and VMAT plans were generated to deliver 60 Gy in 30 fractions to the planning target volume (PTV). For each patient, with the same clinical requirements (target dose prescription, and dose/dose‐volume constraints to organs at risk (OARs)), three plans were designed for sIMRT (five equispaced coplanar beams), IMRT (seven equispaced coplanar beams), and VMAT (two complete arcs). Comparisons were performed for dosimetric parameters of PTV and of OARs (lungs, spinal cord PRV, heart and normal tissue (NT)). All the plans were delivered to a phantom to evaluate the treatment time. The Wilcoxon matched‐pairs, signed‐rank test was used for intragroup comparison. For all patients, compared to sIMRT plans, VMAT plans statistically provide: a) significant improvement in HI and CI for PTV; b) significant decrease in delivery time, lung V20, MLD, heart V30 and spinal cord PRV D1cc; c) significant increase in NT V5; and d) no significant reduction in lung V5, V10, and heart MD. For all patients, compared to IMRT plans, VMAT plans statistically provide: a) significant improvement in CI for PTV; b) significant decrease in delivery time, lung V20, MLD, NT and spinal cord PRV D1cc; c) significant increase in NT V5; and d) no significant reduction in HI for PTV, lung V5, V10, heart V30 and heart MD. For patients with upper esophageal carcinoma, using VMAT significantly reduces the delivery time and the dose to the lungs compared with IMRT, and consequently saves as much treatment time as sIMRT. Considering those significant advantages, compared to sIMRT and IMRT, VMAT is the first choice of radiotherapy techniques for upper esophageal carcinoma.

PACS number: 87.55. D‐

## I. INTRODUCTION

Esophageal carcinoma is one of the most prevalent malignancies and the leading cause of cancer‐related deaths in China. The five ‐year survival rate by radiation alone was comparable to that by surgery for patients with operable upper‐third lesions.^(1)^ Therefore, for diseases located in the upper esophageal region, including cervical and upper thoracic esophagus, radiotherapy is an efficient treatment selection. However radiotherapy treatment planning is challenged by the complex anatomical features of upper esophageal region. The delivery of high radiation dose used in conformal radiotherapy technique is often limited by the tolerance of organs at risk (OARs). Fixed ‐field, intensity‐modulated radiation therapy (IMRT) achieves an even more conformal dose distribution of target structures, while OARs are spared to a greater extent.

High‐modulated IMRT plans using multiple beam angles and complex intensity modulation not only result in a prolonged treatment time, but also reduce the efficiency of radiation because of the use of many small control points.^(2)^ Inefficient radiation leads patients to unnecessary exposure from scatter and leakage dose. Hall and Wuu^(3)^ showed that, compared with the three ‐dimensional (3D) conformal radiation therapy, high‐modulated IMRT caused the increase in volume of normal tissue being exposed to a low‐dose of radiation, which is estimated to increase the incidence of secondary cancers from 1% to 1.75% at ten years. Then simplified intensity‐modulated radiation therapy (sIMRT) technique was widely used in clinic.^(4)^ sIMRT is introduced to reduce the number of MUs, but the HI and CI of the target are deteriorated, as well as the OAR sparing.

In this case, volumetric‐modulated arc therapy (VMAT) was reported as a novel radiation technique, which can achieve highly conformal dose distributions with improved target volume coverage and sparing of normal tissues (NT). Over the past few years, VMAT has been previously compared with IMRT for various types of cancer,^5^, ^6^, ^7^, ^8^, ^9^, ^10^, ^11^, ^12^, ^13^, ^14^, ^15^, ^16^ and several researches have shown that VMAT has the ability to produce dosimetrically equivalent plans to IMRT for centrally located cancers, such as prostate cancer, cervical cancer, anal canal cancer, and head and neck cancers.^4^, ^5^, ^6^, ^7^, ^8^, ^9^, ^10^ In these studies, VMAT was further demonstrated to have the capability of reducing the number of monitor units required to deliver treatments when compared with IMRT, in turn reducing overall treatment times. Martin et al.^(17)^ pointed out that using the arc technique is a good option for mid‐ and distal‐esophageal cancer with primary involvement of the gastroesophageal (GE) junction. However the treatment time hasn't been investigated, and VMAT has seldom been reported in the treatment of upper esophageal carcinoma, which has the complex anatomical features. Therefore, this study aims to evaluate the possibility of using VMAT to integrate the advantages of both sIMRT and IMRT.

## II. MATERIALS AND METHODS

### A. Target volumes and organs at risk

All ten patients in this study were retrospectively replanned for sIMRT, IMRT, and VMAT techniques on Pinnacle^3^ treatment planning systems (TPS) (Philips Healthcare, Andover, MA), which was commissioned according to the TG‐119 recommendations.^(18)^ The gross tumor volume (GTV) and clinical target volume (CTV) were contoured by radiation oncologists. The PTV was delineated with additional 0.5‐1.0 cm margins to the CTV, depending on GTV delineation accuracy and nearby critical structures. Bilateral lungs, heart, and spinal cord PRV, which was generated with a 5 mm margin from spinal cord, were defined as organs at risk (OARs). All anatomical structures were reviewed during chart round. Body excluding the PTV was defined as normal tissue (NT) to quantify the integral dose for each plan. The prescribed dose for this study was 60 Gy in 30 fractions. At least 95% of the PTV received 100% of the prescribed dose.

## B. Plans

For each patient, sIMRT, IMRT, and VMAT plans were designed. The photon beam energy for all plans was 6 MV to be delivered on Elekta Synergy linac (Elekta, Stockholm, Sweden) equipped with 40 pairs of leaves. Dose grid resolution was 0.4×0.4×0.4 cm. For all patients, the beam arrangements of sIMRT and IMRT plans were five and seven equispaced nonopposed coplanar beams in 360°, beginning with 0°, respectively. The number of control point was limited to no more than 25 and 45 for sIMRT and IMRT plans. Therefore, in this article, the difference between sIMRT and IMRT plans was that there were more two beams and dozens of control points in IMRT plans. The beams would be delivered using a multileaf collimator (MLC) with the step‐and‐shoot technique. VMAT plans were generated with two coplanar complete arcs (dual arc). Delivery time was not limited. Continuous gantry motion, dose ‐rate variation, and MLC motion were approximated by optimizing individual beams at 4° gantry angle increments. The same dose‐volume constraints were used for all the plans during inverse planning with direct machine parameter optimization method.^(19)^

Delivery settings for sIMRT (IMRT) were: minimum monitor units for each segment, ten (five); minimum segment area, 10 (5) cm^2^; and maximum number of segments, 25 (45). For VMAT, the gantry angle spacing was 4^0^. The final dose distributions were calculated with the adaptive convolution method. The planning goal was to deliver a prescribed dose of 60 Gy to at least 95% of PTV in 30 fractions; the dose uniformity requirement was ‐5% to +7%. For OARs, V20 for bilateral lungs was no more than 30%, heart V30 was no more than 30%, and the maximum dose delivered to the spinal cord PRV was no more than 45 Gy.

For each pair of sIMRT and VMAT plans, the optimization objectives were the same and originated from IMRT plan. The ultimate goal of optimization for individual patients was that dose to OARs can be kept as low as possible while maintaining optimal target coverage and dose uniformity to the target.

## C. Plan comparison

Following the ICRU Report No. 83, dosimetric parameters were evaluated quantitatively. For the PTV, the parameters were D98% (maximum dose), D2% (minimum dose), mean dose, dose standard deviation, CI, and HI.

CI^(20)^ is defined as follows:

(1) $$\text{CI}=\frac{V_{T,\text{ref}}}{V_{T}}\times \frac{V_{T,\text{ref}}}{V_{\text{ref}}}$$

where $V_{T}$ is the target volume, $V_{T,\mathrm{ref}}$ is the target volume covered by the reference isodose line, and $V_{\mathrm{ref}}$ is the total volume covered by the reference isodose line. The reference dose was 60 Gy. The value of CI is between zero and one. CI=1 represents the ideal situation that the target volume coincides exactly with the treatment volume; CI=0 represents a plan in which there is no overlap between the two volumes.

HI is defined as the difference between the doses covering 5% and 95% of the PTV^(21)^ The equation is as follows:

(2) $$\text{HI}=\frac{D5\%}{D95\%}$$

A greater value of HI indicates a greater degree of dose heterogeneity in the PTV

The dosimetric parameters of OARs were chosen according to OAR characteristics. For bilateral lungs, heart, and NT, the parameters were the mean dose and the percentage volume that was irradiated at specific dose (e.g., V5, V10, and V20 for the lungs). For spinal cord PRV, the parameter D1cc was the maximum dose delivered to $1\;{\text{cm}}^{3}$. Plans were delivered to a phantom for time comparison.

### D. Statistical analysis

The Wilcoxon matched‐pairs, signed‐rank test for nonparametrically distributed data was used to compare VMAT plans with sIMRT plans and sIMAT plans, respectively. The threshold for statistical significance was p<0.05 (two ‐tailed). All statistical analyses were performed using SPSS Version 13.0 (SPSS Inc., Chicago, IL).

## III. RESULTS

### A representative patient

A.

**Figure 1:**
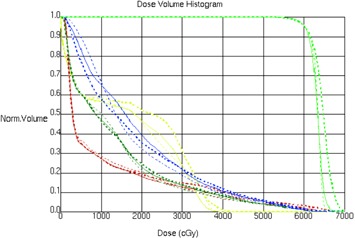
The dose‐volume histogram for PTV (green), lungs (olive green), normal tissue (blue), spinal cord PRV (yellow), and heart (brown) of the three different treatment techniques: IMRT plan (thick dashed line), IMRT plan(thin solid line), and VMAT plan (thin dashed line).

The dose‐volume histogram for planning target volume and OARs of the three different treatment techniques are shown in Fig. 1. The curves show that, when dealing with the low‐dose region (e.g., V5) to the normal tissue, VMAT is worse than sIMRT and IMRT; when dealing with the relatively high‐dose region (e.g., V20), VMAT is superior to sIMRT and IMRT; and when dealing with the high‐dose region (e.g., V64.2), VMAT performs more excellently than sIMRT and IMRT.

**Figure 2:**
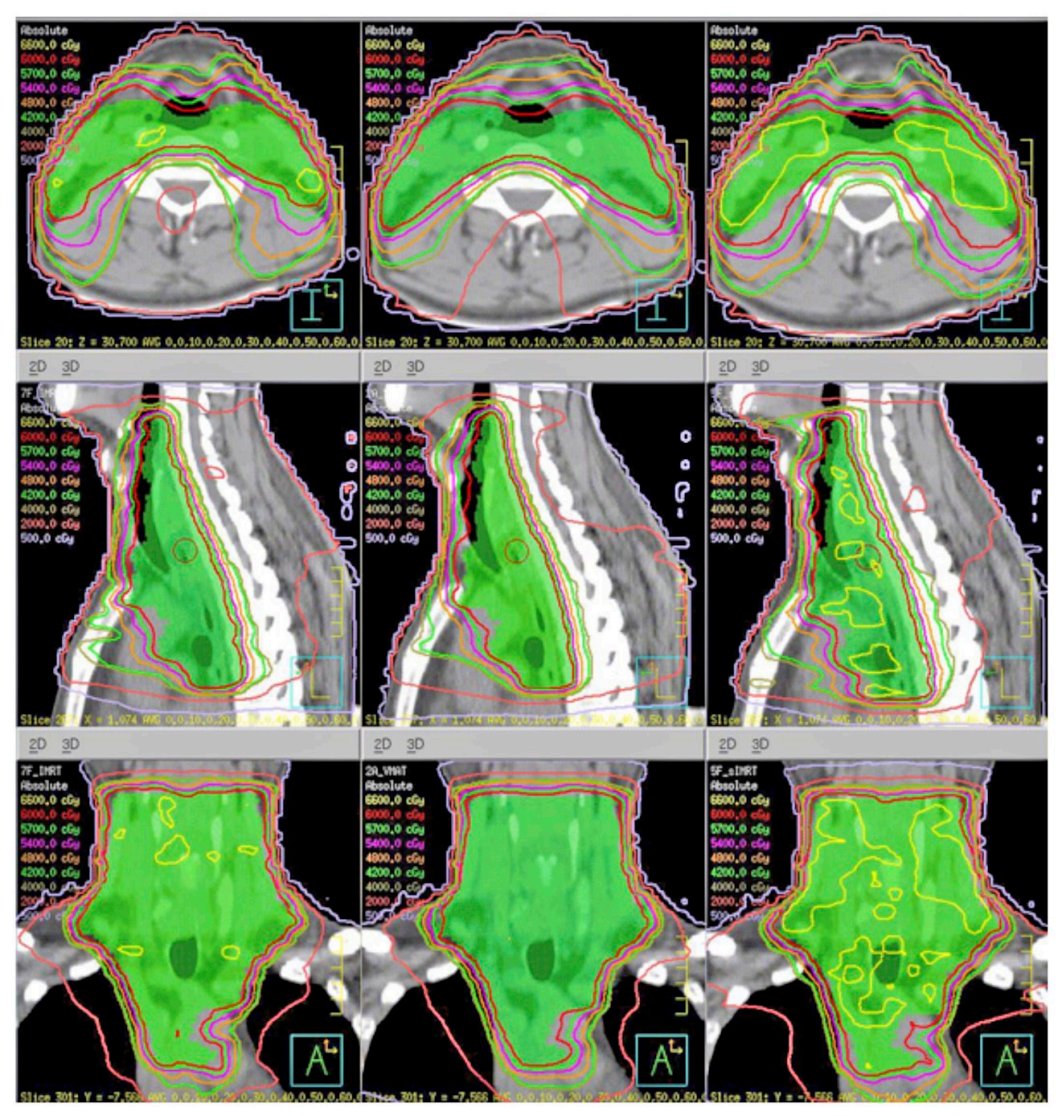
Dose distribution of the three different treatment techniques.

Figure 2 shows the isodose distributions in the central axial, and sagittal and coronal planes for one representative patient. It is obvious that: a) for PTV coverage, PTV homogeneity, and lung V20, the VMAT and IMRT plans are similar and better than the sIMRT plan; b) for spinal cord PRV, the high isodose lines can form bigger C‐shape region to sparing the spinal cord PRV in the central axial planes in VMAT; c) for normal tissue, the 20 Gy isodose line shows significant less volume in the region near to spinal cord PRV in the central axial planes in VMAT; and d) for normal tissue, the 5 Gy isodose line shows significant more volume.

### B. All patients

The results of the study are shown in Table 1 and Table 2. The third column in the table lists the p ‐values. A p ‐value of < 0.05 is considered clinically significant.

**Table 1:** Summary of the study parameters between the sIMRT and VMAT treatment techniques

			*p‐value*
	*sIMRT*	*VMAT*	*sIMRT vs. VMAT*
HI	1.13±0.02	1.09±0.02	0.007
CI	0.84±0.04	0.89±0.02	0.005
D2% (Gy)	68.39±1.10	65.89±0.91	0.005
D98% (Gy)	58.22±0.44	58.55±0.57	0.074
MD (Gy)	64.41±0.48	63.4±0.45	0.005
Delivery Time (min)	5.11±0.38	4.04±0.34	0.005
V5 (lung) (%)	59.86±9.05	59.52±8.45	0.799
V10 (lung) (%)	46.43±6.33	45.85±5.52	0.721
V20 (lung) (%)	23.61±4.23	20.67±3.43	0.005
Mean Lung Dose (Gy)	13.10±2.02	12.65±1.71	0.007
V30 (heart) (%)	8.54±8.52	7.45±7.66	0.018
Mean Heart Dose (Gy)	7.56±4.88	7.53±4.82	0.959
D1cc (spinal cord PRV) (Gy)	43.55±1.26	40.47±1.28	0.005
V5(NT) (%)	73.69±9.70	80.67±11.64	0.005

**Table 2:** Summary of the study parameters between the IMRT and VMAT treatment techniques

			*p‐value*
	*IMRT*	*VMAT*	*IMRT vs. VMAT*
HI	1.10±0.01	1.09±0.02	0.241
CI	0.87±0.03	0.89±0.02	0.007
D2% (Gy)	66.32±0.85	65.89±0.91	0.169
D98% (Gy)	58.43±0.47	58.55±0.57	0.575
MD (Gy)	63.33±0.44	63.4±0.45	0.721
Delivery Time (min)	7.69±0.63	4.04±0.34	0.005
V5 (lung) (%)	60.42±9.24	59.52±8.45	0.059
V10 (lung) (%)	45.47±5.75	45.85±5.52	0.285
V20 (lung) (%)	22.47±3.87	20.67±3.43	0.005
Mean Lung Dose (Gy)	12.87±1.81	12.65±1.71	0.037
V30 (heart) (%)	7.88±8.07	7.45±7.66	0.128
Mean Heart Dose (Gy)	7.53±4.72	7.53±4.82	0.457
D1cc (spinal cord PRV) (Gy)	41.47±0.77	40.47±1.28	0.009
V5(NT) (%)	78.06±10.08	80.67±11.64	0.028

As shown in Table 1, compared to sIMRT plans, VMAT plans statistically provide: a) significant improvement in HI and CI for PTV; b) significant decrease in delivery time, lung V20, MLD, heart V30 and spinal cord PRV D1cc; c) significant increase in NT V5; and d) no significant reduction in lung V5, V10, and heart MD. Therefore, these results indicate that VMAT keeps the advantage of sIMRT and even achieves better, while sparing the OARs and without losing of the HI and CI, but the volume of low‐dose regions for NT is increased.

Compared to IMRT plans (Table 2 provides details), VMAT plans statistically provide: a) significant improvement in CI for PTV; b) significant decrease in delivery time, lung V20, MLD, and spinal cord PRV D1cc; c) significant increase in NT V5; and d) no significant reduction in HI for PTV, heart V30 and heart MD. Consequently, these comparisons show that VMAT keeps the advantage of IMRT and even achieves better, but the volume of low‐dose regions for NT is increased.

## IV. DISCUSSION

In this study, we compared the VMAT with IMRT and sIMRT. The outcome shows that VMAT has the advantage of saving time, even in comparison with sIMRT, which obviously has the advantage of reducing treatment time. With faster treatment, VMAT can improve the machine efficiency, while reducing the discomfort of patients and the possibility of intrafraction movements during treatment.^6^, ^14^ Besides, theoretically, the prolongation of treatment time has negative implication, although the exact clinical effect is still uncertain.^(22)^ Hence VMAT can avoid these minor issues.

For patients with the tumor situated in the paraspinal region, who frequently due to the spinal cord PRV can't suffer the high dose, coverage of the PTV will be worse and even the prescribed dose will be decreased. However, VMAT indicates stronger ability to control the distribution of high‐dose area through intensity modulation and gantry continuous rotation.^(6)^ Therefore, the oncologists can scale up the delivered dose to tumor and provide a potential improvement in treatment outcome.^(23)^

In this work, we also investigated the planning time (data not shown). Compared with IMRT, the time per iteration optimization and per final dose computation for VMAT is three and 20 times larger, respectively. Totally, the planning time will be prolonged by nearly five times. And it's reported that the increased planning time limits the VMAT clinical application.^6^, ^8^, ^12^ However, Quan et al.^(24)^ developed an AutoPlan system to automatically generate the VMAT plans with less planning time. In future, more advanced optimization system may emerge to reduce treatment planning time.^(25)^

The results also indicate that VMAT plans perform better in dosimetric parameter indices (V20 and mean lung dose) than sIMRT and IMRT using the same dose constraints. Although the V5 and V10 in VMAT plans were not significantly higher than those in IMRT plans, obviously they got increased when compared to conformal plans,^26^, ^27^ which may lead to increase the risk of radiation pneumonitis. Hall and Wuu^(3)^ concluded that IMRT may approximately double the risk of secondary cancers as a consequence of the changing CRT to IMRT because of a bigger volume of normal tissue being exposed to lower doses. Our finding agrees with the conclusion of Quan et al.^(24)^ that VMAT creates a larger volume of low‐dose irradiation to normal tissue than IMRT. In order to learn more about the feature of the low‐dose region in VMAT plans, dose volume constraints V5 were assigned to the lungs. The results show the volume of low‐dose regions could be limited using VMAT. In addition, the analysis shows that the HI of VMAT plans is superior to IMRT plans, which has never been reported before, and this may be attributable to the numerous beam angles, especially in the region of heterogeneous electron density. Further investigation is required to confirm the inference.

## I. CONCLUSIONS

Our study demonstrates that, for patients with upper esophageal carcinoma, using VMAT significantly reduces the delivery time and the dose to the lungs compared with IMRT and, consequently, saves as much treatment time as sIMRT. Considering those significant advantages, compared to sIMRT and IMRT, VMAT is the first choice of radiotherapy techniques for upper esophageal carcinoma.
